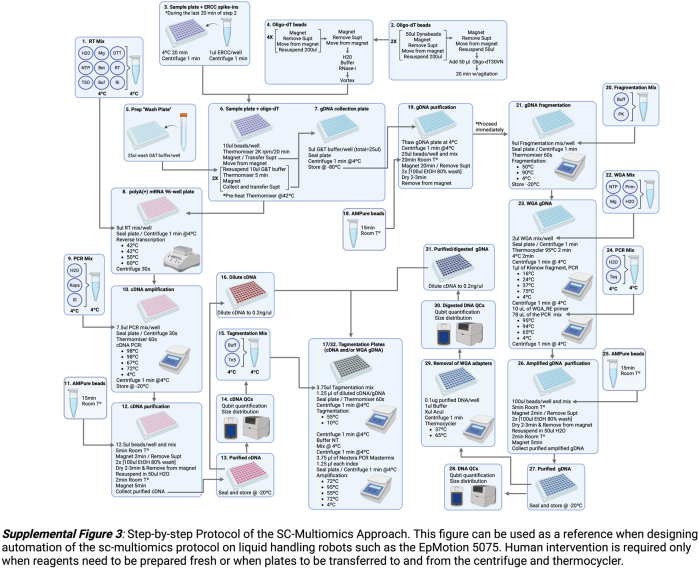# A single-cell multiomics approach for simultaneous analysis of replication timing and gene expression

**DOI:** 10.1016/j.jbc.2025.110581

**Published:** 2025-08-08

**Authors:** Anala V. Shetty, Clifford J. Steer, Walter C. Low

**Affiliations:** 1 Molecular, Cellular, Developmental Biology, and Genetics Graduate Program, University of Minnesota, Minneapolis, Minnesota, USA; 2 Stem Cell Institute, University of Minnesota, Minneapolis, Minnesota, USA; 3 Department of Neurosurgery, University of Minnesota, Minneapolis, Minnesota, USA; 4 Division of Gastroenterology, Hepatology and Nutrition, Department of Medicine, University of Minnesota, Minneapolis, Minnesota, USA

**Keywords:** DNA replication, gene expression, replication timing, single-cell multiomics, single-cell sequencing, single-cell protocols

## Abstract

Replication timing (RT) allows us to analyze temporal patterns of genome-wide replication, that is, if genes replicate early or late during the S-phase of the cell cycle. RT has been linked to gene expression in normal and diseased acute and chronic states, such as cancer. However, studies done to date focused on bulk cell populations that required tens of thousands of cells for RT analysis. Here, we developed an affordable novel single-cell (sc) multiomics approach to simultaneously analyze RT and gene expression from cells or nuclei. We used this approach to generate single-cell RT profiles and sc-gene expression data from the well-established human liver cancer cell line, HepG2. We demonstrated that as few as 17 mid S-phase cells were sufficient to produce cell type–specific pseudo bulk RT profiles that had a high correlation to previously published HepG2 bulk RT profiles. The single-cell RT profiles allowed us to visualize how individual cells progressed through genome replication. We were also able to demonstrate cell-specific correlations between RT and gene expression, which to our knowledge, has not been reported. We observed trends that were conserved between individual cells, as well as cell-to-cell variations, which were not possible to detect with the bulk RT studies.

Replication timing (RT) of cells provides insight into how different parts of the genome replicate during S-phase of the cell cycle. We can deduce if individual genes replicate during the early, mid, or late S phases of the cell cycle. RT is conserved within cell types, is mitotically inherited (11), and is regulated in megabase–sized bins throughout the genome (26). RT of the genomic DNA (gDNA) is disrupted in diseased states, such as premature aging (28), cancer (17), and other acute and chronic conditions. Multiple cancer studies have shown that alterations in RT vary from small location-specific RT changes to larger chromosome wide delays in RT, as observed in 80% of cancers over the last century (7, 33). Although RT is affected in cancer cells, the cause or consequence for altered RT is not clear (3).

RT has also been correlated with gene expression in both normal and diseased states. Previous studies have demonstrated that genes in early replicating domains are usually expressed, and genes in late replicating domains have low gene expression or are not transcribed (13, 27). The ability to study RT and its associations with gene expression, at the level of individual cells, would be powerful in understanding how these multilayer controls exist in normal and diseased cells.

We developed a novel single-cell multiomics approach that enables simultaneous analysis of RT and the accompanying transcriptome, from the same single cells, which to our knowledge, has not been reported in the literature. This allows us to observe cell-to-cell variation in RT and gene expression as well as to perform RT-gene expression correlations within individual cells. For this study, we chose a well-established human HepG2 cancer cell line (15). HepG2 is a model cell line for studying human hepatocellular carcinoma cancer as well as primary hepatocytes *in* vitro (32). These cells are easy to grow, and our laboratory had previously generated bulk RT data for HepG2 cells (21). In processing HepG2 cells using the single-cell multiomics protocol, we analyzed (i) genome-wide coverage and copy number variations (CNVs); (ii) single-cell RT (sc-RT) and gene expression; and (iii) correlation between RT and gene expression using pseudo bulk profiles and within individual cells. For the first time, we demonstrated RT-gene expression correlations within the same cells and at the single-cell level. The results of our study emphasize the importance of the sc-multiomics approach in capturing cell-specific relationships and associations between the gDNA and mRNA.

## Results

### Development of the single-cell multiomics protocol

Protocols for generating either sc-RT or sc-RNA profiles exist, but simultaneous analysis of both parameters, from the same cells, has not been reported. Single-cell multiomics techniques for extraction of both DNA and RNA have been previously published. Although some of these techniques employ good strategies for separation of sc-DNA from sc-RNA per cell (6, 19), they use commercial kits for downstream amplification of the nucleic acids and are not compatible for processing nuclei acids from nuclei (instead of cells) as the starting material (9) or use barcoding techniques that were optimized for tens of thousands of cells (34).

Advantages of our in-house sc-multiomics protocol include (i) compatibility with both cells and nuclei; (ii) compatibility with very small cell numbers (as low as 10–20 cells) that can be used to process both genome and transcriptome per cell from scarce patient samples/limited cell numbers; and (iii) affordability *versus* commercially available kits (∼15% of the cost of commercial kits for processing sc-gDNA and scRNA).

We were inspired by protocols like the G&T-Seq (18), which process genome and transcriptome from cells, and Smart-Seq2 technique (25), and adopted/combined their strategies for separating gDNA and mRNA from each cell, and for processing mRNA. For amplification of gDNA, we developed an affordable in-house gDNA protocol, which can also be used as a stand-alone protocol. This gDNA protocol is more compatible with the newer two-color chemistry Illumina sequencing platforms, such a NovaSeq and NextSeq, compared with previously published in-house gDNA protocol (2); and it significantly reduces sequencing depth per sample for generating sc-RT profiles. This protocol can be used for detecting sc-RT and sc-CNV at the level of individual genes. The sc-gDNA processed using this protocol was compatible with two commonly used computational sc-RT pipelines, the Kronos sc-RT pipeline (8) and the scRepli-Seq pipeline (23).

Bulk RT protocols generally use 20,000 to 40,000 cells per replicate of the G1, early S, late S, and G2 populations (with three replicates per experiment) (20). With the in-house protocol, we demonstrated that as few as 17 single HepG2 mid S-phase nuclei/cells were sufficient to deduce pseudo bulk RT profiles that demonstrated high correlation with the bulk RT profile. This demonstrated the unique ability of the current protocol to drastically reduce cell numbers, while providing high-resolution cell-specific and gene-specific information.

#### Separation of gDNA and mRNA from single cells

**Figure 1:**
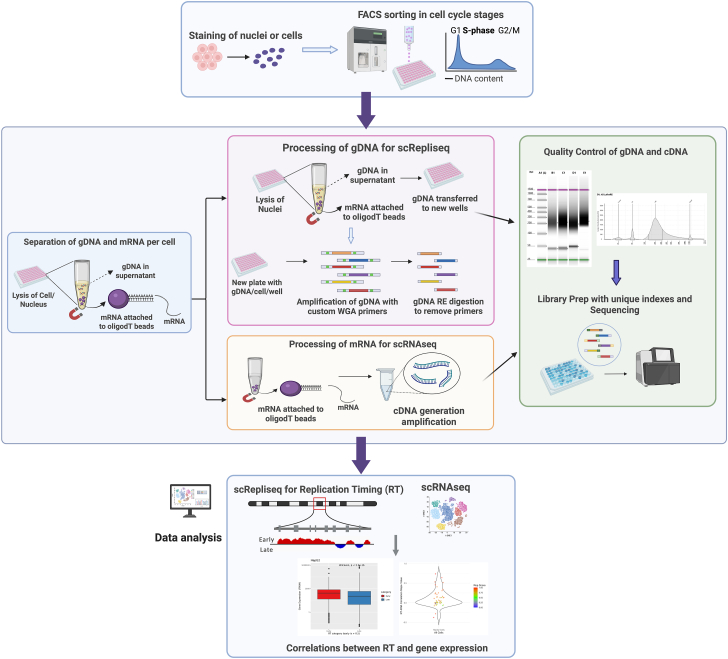
**Single-cell multiomics approach for determining replication timing (RT) and gene expression.** Single cells (or nuclei) were stained with a DNA dye and then sorted into 96-well plates, based on their cell cycle. From each individual cell, both gDNA and mRNA were isolated, separated, and amplified using in-house protocols. Quality control of amplified gDNA and cDNA (derived from mRNA) was performed to ensure successful amplification, after which the samples were library prepped for sequencing. After sequencing, data analysis was performed to obtain information regarding copy number variation, RT, and gene expression from each cell. High-resolution correlations between RT and gene expression were drawn within each cell. cDNA, complementary DNA; gDNA, genomic DNA.

The detailed steps for separation of gDNA from mRNA within each cell are described in the Experimental procedures section. The separation of gDNA from mRNA was adopted from the G&T protocol with minor modifications (19). Briefly, single cells/nuclei were sorted into RLT buffer in 96-well plates, with one cell per well. The single cells disintegrated in the RLT buffer releasing the gDNA and the RNA into the solution. The mRNA in each well was pulled down using the Smart-Seq2 technique of using poly-dT tailed primers attached to magnetic beads (25). The poly-dT primers were complementary to the poly-A tails of the mRNA. The beads were pulled down using a low elution magnet, leaving the gDNA in the supernatant (Fig. 1). The supernatant containing the gDNA was then separated into a new 96-well plate, maintaining the cell identity per well.

#### Amplification of single-cell gDNA using an *in-house* protocol

**Supplemental Figure 1:**
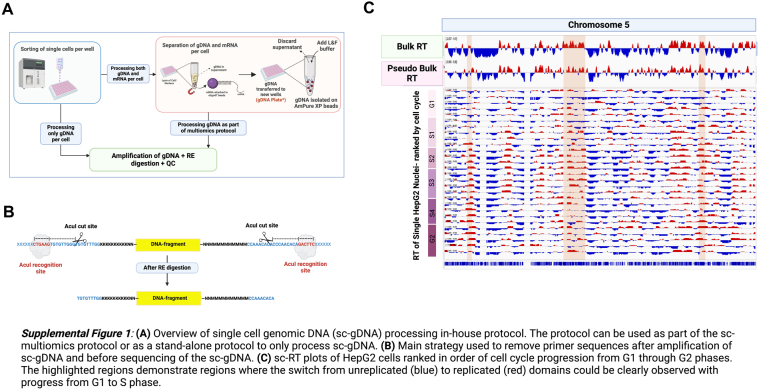


We developed an in-house protocol for processing sc-gDNA from either cells or nuclei. This protocol can be used for processing only gDNA from samples or can be used as part of the multiomics protocol. If performing the multiomics approach, we first pulled down the gDNA from the gDNA supernatant onto AMPure XP beads (Fig. S1*A*). When processing only gDNA, cells/nuclei were directly sorted into the L&F buffer and processed. In both cases (with or without beads), we followed the same subsequent processing steps as described in the Experimental procedures section. We used a degenerate oligonucleotide primed-PCR technique for amplification. We designed wheat germ agglutinin oligos that contained restriction enzyme (RE) digestion sites specific to the enzyme AcuI. After gDNA amplification, we performed RE digestion to remove the majority of primer sequences from both ends of the amplified DNA (Fig. S1*A*). This drastically reduced the presence of the high G% and repeat regions in the final gDNA fragments of interest.

This protocol is more compatible with the newer Illumina sequencing machines that use two-color chemistry (*e.g.*, NextSeq, NovaSeq), compared with the Bartlett protocol (2), which was only compatible with the four-color chemistry sequencing platforms (*e.g.*, HiSeq [discontinued], MiSeq).

The two-color sequencing machines have filters that automatically discard low diversity sequences, such as the high % “G” repeat regions on the wheat germ agglutinin primers. Sequencing of the gDNA fragments without RE digestion led to the machine automatically discarding the majority of reads, because of the low diversity primers in the first 25 cycles. This issue was observed when sequencing gDNA processed using the Bartlett protocol (2). This was addressed and resolved by using the RE digestion step in the protocol.

#### Amplification of single-cell mRNA

After the separation step using poly(dT)-tailed magnetic beads, the mRNA was first converted into complementary DNA (cDNA) as described in the Experimental procedures section. Reverse transcription and amplification of mRNA was performed in-house using the strategy in G&T with minor modifications (19). We performed quality control (QC) to ensure that the amplified cDNA was in the size range expected before tagmentation-based library preparation as described.

### Processing HepG2 nuclei using the single-cell multiomics protocol

#### Sorting HepG2 nuclei based on cell cycle phase

**Figure 2:**
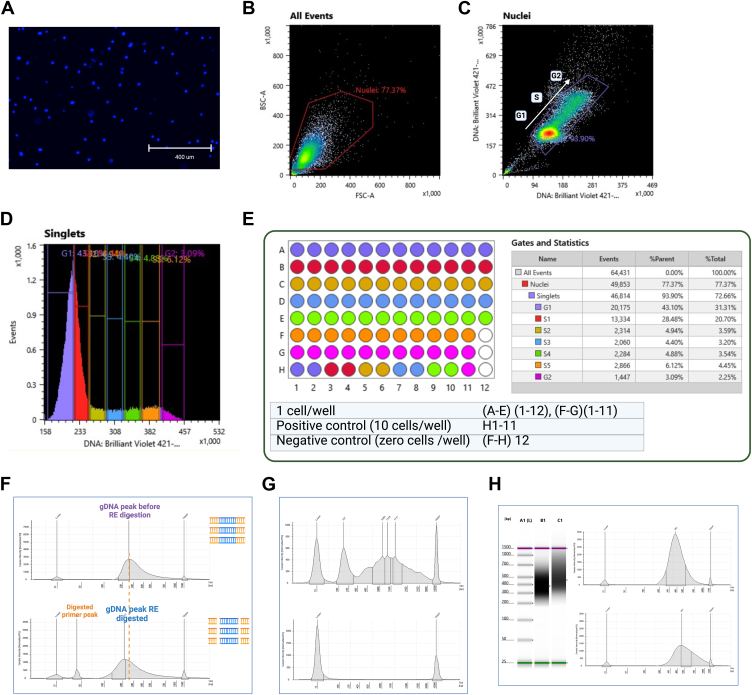
**Sorting of stained HepG2 for the single-cell multiomics protocol according to the cell cycle.***A*, HepG2 nuclei were stained with the DNA-specific dye, Vybrant DyeCycle Violet. Sorting gate to capture (*B*) all events (which includes all HepG2 nuclei) and (*C*) to capture stained nuclei in the G1, S, and G2 phases of cell cycle. These were determined by the level staining of DNA dye on *X*-axis and *Y*-axis. *D*, all events on *Y*-axis with DNA-specific staining on *X*-axis. The sorting gates for G1, S, and G2 phase cells were set as shown. The S phase was divided into five equal gates from S1 to S5. The identity of each gate is shown in the *right panel* in (*E*). *E*, example of the design of 96-well plate sorted for the sc-multiomics protocol. *Left panel,* design of a 96-well sorted plate. Single cells and positive and negative control wells were included. *Right panel,* gates sorted and colors assigned to each gate. Statistics of total events in each gate. *F*, amplified gDNA peaks before (*top panel*) and after RE digestion (*bottom panel*) detected using a Bioanalyzer, on a high-sensitivity D1000 ScreenTape. Shift in the gDNA peak after RE digestion and appearance of the digested primer peak confirmed successful RE digestion. *G*, amplified cDNA peak detected using a Bioanalyzer, on a high-sensitivity D5000 ScreenTape. The *top panel* demonstrates successful amplification of single-cell cDNA with multiple peaks at 1.5 to 2 kbp. The *lower panel* shows a negative control well, which had no nucleus. *H*, pooled gDNA and pooled cDNA wells were observed using a high-sensitivity D1000 ScreenTape after library preparation and before sequencing. A1 is the ladder, B1 is the pooled gDNA samples, and C1 is the pooled cDNA samples. *Top right panel,* the pooled gDNA peak (B1) was observed at 382 bp. *Bottom right panel,* pooled cDNA peak (C1) was observed at 461 bp. RE, restriction enzyme.

For the multiomics protocol, we processed HepG2 nuclei. We isolated nuclei from cells and sorted them based on DNA content using a previously optimized in-house protocol (21). We stained the isolated nuclei with Vybrant DyeCycle Violet, which is a DNA-specific dye (Fig. 2*A*). This allowed us to sort cells from specific phases of the cell cycle, based on DNA content (Fig. 2*C*) from the total population (Fig. 2*B*). Cells in the G1 phase have two copies of chromosomes, cells in G2 have four copies, and the population in between is in the S phase, where the cells are actively replicating (>2 copies but <4 copies).

When performing bulk Repli-Seq, the S phase is sorted into “early” and “late” S-phase populations (30). With the single-cell in-house approach, we gated the S phase population into five gates, S1 through S5 (Fig. 2*D*). This allowed us to track the gradual progression of genome replication through the S phase. For this study, we processed a total of 48 HepG2 nuclei across different cell cycle phases (13 G1 phase cells, 29 S1–S5 phase cells, and 6 G2 phase cells) as described in the Experimental procedures section. The sorting design and cell cycle phase identity of each well is shown in Figure 2*E*. We also sorted for and processed positive and negative control wells.

Throughout the multiomics protocol, QC was performed at regular intervals to ensure that no sample loss or contamination occurred. This also ensured successful amplification and processing of gDNA and cDNA at the different steps.

#### QC of amplified gDNA

We quantified the HepG2 gDNA per well using a Qubit. We obtained between 750 and 1000 ng of amplified gDNA per cell for 27 PCR amplification cycles. We also visualized amplified gDNA samples before and after RE digestion using high-sensitivity screen tapes on the Bioanalyzer. The amplified gDNA band was observed between 200 and 500 bp with a peak around 250 bp (Fig. 2*F*, *top panel*). After successful RE digestion, we observed a digested primer peak ∼45 bp along with a decrease in the gDNA fragment peak (from ∼250 to ∼205) (Fig. 2*F*, *bottom panel*). The absence of the primer peak at ∼45 bp indicated that RE digestion was not successful.

#### QC of amplified cDNA

We quantified the amplified cDNA using a Qubit. We found that 22 to 24 amplification cycles were ideal for HepG2 cDNA amplification. We obtained 20 to 30 ng (22 cycles) and 75 to 100 ng (24 cycles) of cDNA per cell. We also visualized the amplified cDNA fragments on the Bioanalyzer. We observed a broad cDNA band between 500 and 4000 bp with multiple peaks around 1.5 to 2 kbp (Fig. 2*G*, *top panel*). The Smart-Seq2 technique pulled down and amplified full-length mRNAs of different lengths (25). No peaks were observed in the cDNA negative control well (no nucleus) (Fig. 2*G*, *bottom panel*). Occasionally, we observed a peak around 150 bp, specific to the amplified primers. However, the bead purification steps after cDNA library preparation removed these primers.

We used a tagmentation-based library preparation kit for the amplified gDNA and cDNA, as described in the Experimental procedures section. We also employed uniquely barcoded oligos for gDNA and cDNA libraries, performed QC of the library prepped gDNA and cDNA (Fig. 2*H*), before pooling them for sequencing.

### CNV and coverage in HepG2 single-cell gDNA

**Figure 3:**
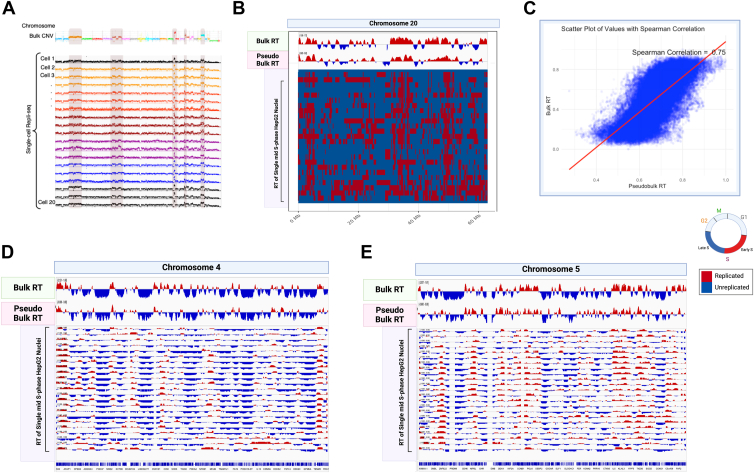
**HepG2 single-cell replication timing (RT) profiles are similar to HepG2 bulk RT profile.***A*, HepG2 genome coverage for bulk (*top panel*) and single-cell gDNA was generated from the multiomics protocol. CNVs in chromosomes 2, 6, 14, 16, and 20 are highlighted in the figure. *B*, HepG2 bulk, pseudo bulk of sc-RT profiles, and binarized sc-RT profiles for chromosome 20 are shown. Each row in the binarized plot is a S-phase single cell. *C*, Spearman's correlation of 0.75 was observed between genome-wide HepG2 bulk RT and pseudo bulk RT derived from sc-RT profiles. For (*D*) chromosome 4 and (*E*) chromosome 5, HepG2 bulk, pseudo bulk, and nonbinarized sc-RT profiles are provided. Replicated regions are highlighted in *red*, and nonreplicated regions are highlighted in *blue*. CNV, copy number variation; gDNA, genomic DNA; sc-RT, single-cell RT.

We observed good genome coverage for all the sequenced sc-gDNA samples, comparable to the reference bulk HepG2 coverage (Fig. 3*A*). Previously sequenced bulk HepG2 genome demonstrated aneuploidy in chromosomes 2, 6, 16, 17, and 20. Similar to the bulk genome, we observed chromosome-specific CNV in the single-cell genome plots (Fig. 3*A*). We also identified genes with CNV that were conserved across the HepG2 cells. These genes included TP53 binding protein, *TP53BP2* (14); proto-oncogene, *MYC* (4); Wnt regulator, *CTNNB1* (10); PI3K pathway component, *PIK3AP1* (1); MAPK/ERK pathway kinase, *BRAF* (5); and tumor suppressors, *CDK2A*, *CDK2B*, and *CDK2C* (36), all of which are known to be mutated in the HepG2 cancer cells. A more complete listing of genes with CNV can be found in the attached Supplemental file 1.

### Analysis of sc-RT in HepG2 cells

We used established computational pipelines—Kronos sc-RT (8) and the sc-Repli-Seq (23) for generating HepG2 sc-RT profiles, as described in the Experimental procedures section. For identifying sc-RT profiles, genome wide reads of S phase cells were normalized against G1 phase control cells. Since G1 cells have two copies of chromosomes, and G2 cells have four copies of chromosomes, these were used to detect genome-wide CNV in the S phase cells. For analysis *via* the sc-Repli-Seq pipeline, we assigned each HepG2 cell to its respective cell cycle phase (G1, S, or G2), based on sorting parameters. The Kronos analysis pipeline not only is designed to bioinformatically detect cell cycle phases of cells based on reads across the genome but also has an option to assign cells to specific known cycle phases. The sc-RT profiles revealed conserved early or late replication domains across all cells, while also demonstrating smaller cell-to-cell variations. This could be observed in the binarized plot for chromosome 20 (Fig. 3*B*) and in the nonbinarized sc-RT plots for chromosome 4 and 5 (Fig. 3, *D* and *E*). Each row represents a sc-RT profile. Common patterns of replicated domains (red bins) were observed across all the cells. We also generated a HepG2 “pseudo bulk” plot by combining the HepG2 sc-RT profiles of 17 single cells in the mid S-phase, that is, cells sorted from S2 (n = 6), S3 (n = 5), and S4 (n = 6) sorting gates (Fig. 2, *D* and *E*), as described in the Experimental procedures section. We excluded cells from the early S phase (S1 gate) and late S phase (S5 gate). Cells in the mid S-phase are ideal for generating the pseudo bulk plot (8) as a clear distinction between early and late replicating regions can be observed. In the mid S phase, ∼50% of the cell’s genome is replicated (assigned as “early” replicating in *red* in Fig. 3, *B*, *D*, *E*), and the remaining ∼50% is not replicated (“late” replicating in *blue*). The HepG2 pseudo bulk profile generated from the 17 mid-S phase single cells had a high correlation (correlation coefficient = 0.75) to the bulk reference RT profile generated from tens of thousands of S-phase cells (Fig. 3*C*). This high correlation could also be visually observed in Figure 3, *B*, *D*, *E*, where patterns of red and blue were conserved between the bulk and the pseudo bulk profiles for chromosomes 4, 5, and 20.

Previous scRT protocols used ∼130 to 150 mid S-phase cells to deduce pseudo bulk RT (8, 22, 23). Here, we demonstrated that with the gDNA processed using the in-house protocol, we could achieve significantly lower cell numbers. Since we sorted cells from the G1 phase, we were also able to observe the sc-RT domain switch from unreplicated to replicated, as cells transitioned from G1 to S phase (highlighted in Fig. S1*C*).

### Gene expression of HepG2 nuclei

**Figure 4:**
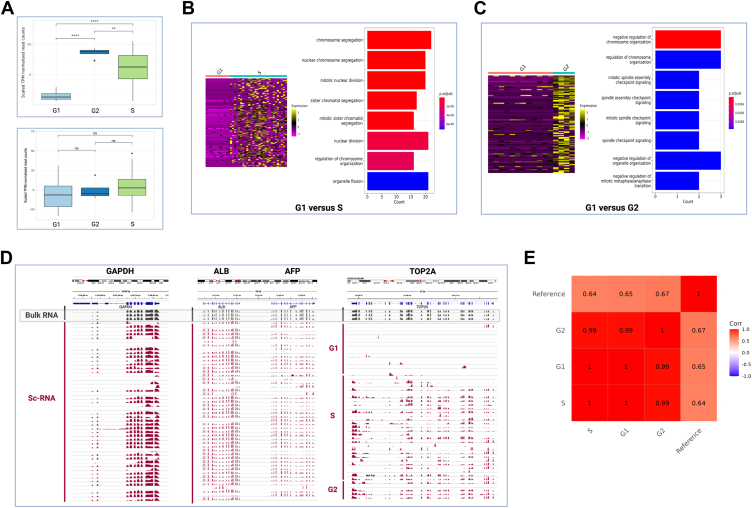
**Gene expression analysis of scRNA derived using the multiomics protocol.***A*, level of expression of G2 phase–specific genes (*top panel*) and S phase–specific genes (*bottom panel*) in populations of sorted 13 G1-, 29 S-, and 6 G2-specific HepG2 cells. *B*, top differentially expressed genes (DEGs) between G1 and S cells and GO terms of top DEGs. *C*, top DEGs between G1 and G2 cells and GO terms of the top DEGs. Significance was calculated using the pairwise *t* test between phase groups with a *p* ≤ 0.05 cutoff for (*B*) and (*C*). *D*, comparison of gene expression between HepG2 bulk profiles and sc-RNA profiles. *GAPDH*, *ALB*, and *AFP* were expressed across all populations. *TOP2A* was expressed in bulk, S, and G2 cells but was not expressed in G1 cells. *E*, correlation between HepG2 scRNA from G1, S, and G2 phases with RNA-Seq reference dataset (GSM923446). DEG, differently expressed gene; GO, Gene Ontology.

Using the sc-multiomics protocol, we processed and analyzed mRNA from the same 48 HepG2 cells used for sc-RT analysis. We detected an average of 5586 unique genes per cell. We analyzed expression of cell cycle stage–specific genes in the HepG2 cells sorted from G1, S, and G2 phases. G2/M-specific genes were significantly upregulated in the G2 cells compared with G1 and S phase cells (Fig. 4*A*, *top panel*). We did not see statistically significant differences in S-phase-specific genes between cells from the three cell cycle phases (Fig. 4*A*, *bottom panel*). We hypothesized that S-phase-specific genes begin upregulating expression toward the end of G1 phase. Further, the S-phase transcripts may still be present after S phase is completed, that is, as cells move into the G2 phase. Hence, even in G2, the cells may still contain S-phase-specific transcripts. These factors could be contributing to the lack of significant differences in S-phase-specific genes as observed in Figure 4*A*.

We also generated heatmaps and analyzed Gene Ontology (GO) terms of the top 100 differentially expressed genes (DEGs) between stages, as described in the Experimental procedures section. Top DEGs between G1 and S cells were associated with aspects of chromosome segregation and chromosome organization, which are relevant as the cell transitioned from the G1 phase to DNA synthesis in the S phase (Fig. 4*B*). GO terms for G1 *versus* G2 phase were associated with chromosome organization and aspects of mitotic spindle checkpoint signaling (Fig. 4*C*). These analyses confirmed that the sc-RNA processing protocol allows us to capture a large number of unique genes and conserved cell cycle stage–specific gene expression.

**Supplemental Figure 2:**
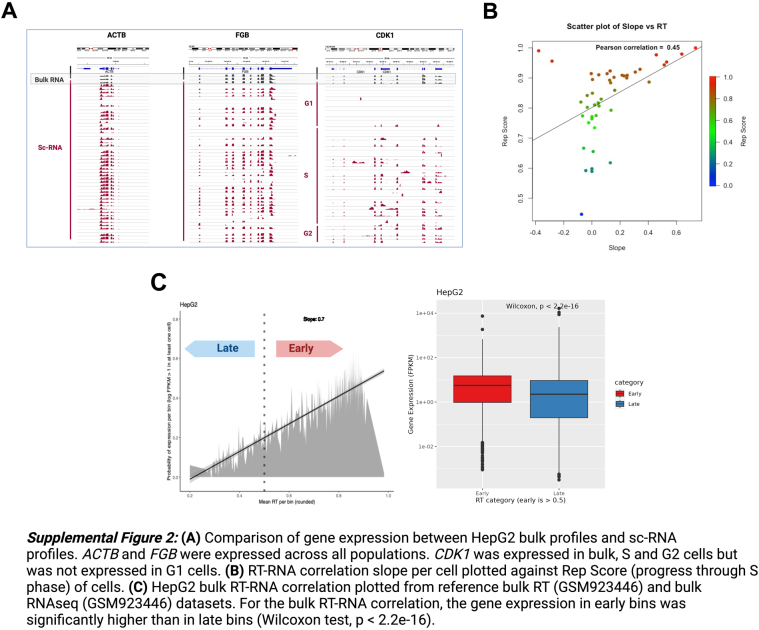


We observed high correlation between our HepG2 sc-RNA data and reference bulk HepG2 RNA-Seq dataset (Fig. 4*E*). We also compared housekeeping genes, hepatocyte-specific genes, and cell cycle–specific genes between the sc-RNA and bulk expression profiles (Figs. 4*D* and S2*A*). Expression of housekeeping genes like *GAPDH* and beta-actin gene (*ACTB*), as well as hepatocyte-specific genes, like albumin (*ALB*), alpha fetoprotein (*AFP*), and fibrinogen (*FGB*), was conserved between the sc-RNA profiles and the bulk RNA profiles (Figs. 4*D* and S2*A*). We also determined expression of cell cycle stage–specific genes, cyclin-dependent kinase 1 (*CDK1*) and DNA topoisomerase II alpha (*TOP2A*). *CDK1* is highly expressed in G2/M phase but has very low or no expression in G1 phase (16). *TOP2A* is a proliferation marker that is expressed in the S/G2/M phases but has low expression or is absent in the G1 phase (24). Expression of *CDK1* and *TOP2A* was limited to single cells in the S and G2 phases only. No expression was observed in the G1 phase cells (Figs. 4*D* and S2*A*).

### Correlation between RT and gene expression

**Figure 5:**
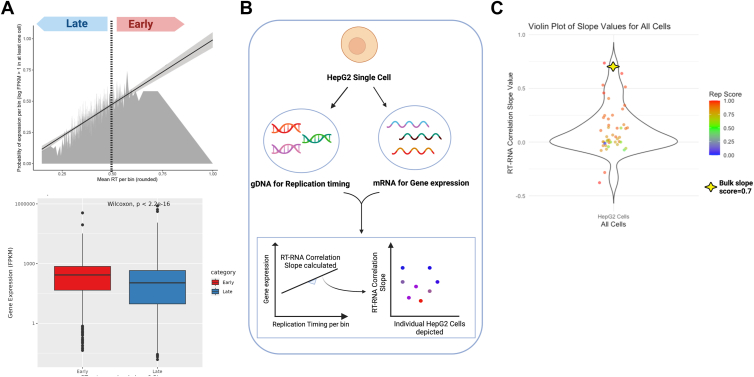
**HepG2 replication timing (RT) and gene expression (RNA) correlations at pseudo bulk and individual cell level.***A, top panel,* pseudo bulk RT-RNA correlation plot with correlation slope. Bins are divided into early (≥0.5) and late replicating bins (<0.5). *Bottom panel,* significantly higher gene expression was observed in the early bins compared with late bins in the pseudo bulk RT-RNA analysis (Wilcoxon test, *p* < 2.2e-16). *B*, method used for deriving RT-RNA correlations within individual cells. Within each cell, the gene expression was plotted against RT and the slope was calculated. *C*, the correlation slopes of individual cells were then plotted. Cells were colored by “Rep Score,” which represents the cells’ progression through S phase (*e.g.*, Rep score = 0.6 means 60% of the genome has been replicated). The RT-RNA slope value derived from the bulk RT-RNA correlation is marked in the plot.

Since we simultaneously extracted both gDNA and mRNA from the same cells, this enabled us to perform high-resolution correlations between RT and gene expression (RT-RNA). Previous studies performed in bulk populations have analyzed correlations between RT and gene expression in cells from different species, such as mouse (12, 13), *drosophila* (31), and human (27, 29). These bulk studies found that early RT domains had higher probability of gene expression compared with late replicating domains. Previous studies also demonstrated that early replication domains tended to have higher chromosome accessibility compared with late replicating domains, which then would allow access to transcription machinery for gene expression in the early replicating domains (29). However, these correlation studies used separate cohort of cells for extraction of gDNA and RNA. Further, sc-RT-RNA correlation studies, with gDNA and RNA extracted from the same cells, have not been reported to date. We applied a similar strategy used by the above-described studies, for calculating the RT-RNA correlations, as described in the Experimental procedures section. Briefly, we calculated the probability of gene expression for all genes across the genome (that is, on or off) and plotted the probability of that expression with gene-specific RT. We then calculated the slope value and gene expression probability in early *versus* late domains (Fig. 5*A*).

Here, we performed RT-RNA correlations using (i) pseudo bulk RT and RNA for population trend and (ii) sc-RT and sc-RNA for individual cells’ trends. We observed a positive slope correlation between RT and RNA in the pseudo bulk analysis (Fig. 5*A*, *top panel*). Early replicating bins (>0.5) had significantly higher gene expression than late replicating bins (≤0.5) in the pseudo bulk RT-RNA analysis (Fig. 5*A*, *bottom panel*). We also generated the bulk HepG2 RT-RNA plot from reference RT and RNA datasets and observed a similar positive slope trend (Fig. S2*C*).

Next, we analyzed RT-RNA correlations within individual cells. Briefly, for each HepG2 single cell, we calculated the RT-RNA correlation slope value using cell-specific RT and gene expression (Fig. 5*B*). We then plotted the slope values in the violin plot in Figure 5*C*, where each dot represents a single HepG2 cell. We colored cells based on their progression through S phase, as denoted by “Rep Score” in Figure 5*C*, with a Rep Score of 0.6 indicating 60% of the cell’s genome has been replicated.

We observed that the majority of cells had a positive RT-RNA correlation slope value (slope >0), showing a positive trend similar to the pseudo bulk and bulk RT-RNA positives (Fig. 5*C*). This suggests that, at the single-cell level, early replicating RT domains tend to have higher levels of gene expression when compared with the late domains. However, while the bulk RT-RNA slope was = 0.7 (Fig. 5*C*), the individual cell scores varied cell to cell. Most of the cells had a lower correlation score close to 0, which suggested that there was a low positive correlation between RT and gene expression in majority of the cells. We chose the nonparametric Wilcoxon signed-rank test to validate our hypothesis that the median of our cells’ RT-RNA slope values was significantly different from the condition where there was no RT-RNA correlation, that is, RT-RNA slope = 0. We obtained a *p* value of 0.001137, supporting our hypothesis.

Next, we wanted to observe if the correlation between RT and gene expression changes as cells progress through S phase, that is, if the RT-RNA slope value changes with Rep score of the cells. Hence, we plotted RT-RNA slope values against the Rep scores and observed a moderate positive correlation (Pearson's correlation coefficient = 0.45) between the two variables (Fig. S2*B*). As the cells progressed through S phase, they tended to have a higher correlation between RT and gene expression, especially for cells with Rep score >0.6 (Fig. S2*B*). Overall, using the sc-multiomics approach, we were able to capture sc-RT-RNA correlations and observe how they changed through S phase. These cell-to-cell variations were lost with the pseudo bulk and bulk analyses. Future studies can focus on generating more datapoints within the same cell line, studying the different subpopulations based on RT-RNA scores, and analyzing how gene expression might vary between them.

## Discussion

We have developed an in-house single-cell multiomics protocol to simultaneously analyze RT and gene expression, at the single-cell level. This in-house protocol is much more affordable than the combined costs of commercially available kits for processing sc-gDNA and sc-RNA (∼15% of the kits’ costs for every 500 cells). Using this multiomics protocol, we extracted and amplified gDNA and mRNA from HepG2 nuclei. We analyzed genome coverage and observed CNVs at the chromosomal and gene levels. We also derived sc-RT profiles from gDNA and sc-gene expression information from mRNA. The gDNA extracted and amplified using this in-house protocol was compatible with both commonly used sc-RT pipelines, Kronos (Fig. 3*B*) and sc-Repli-Seq (Fig. 3, *D* and *E*). Most importantly, we observed cell-specific RT, across the different stages of the S phase.

In cancer cells, changes in RT have been associated with aberrant gene expression and epigenetic changes (7). Future studies can interrogate specific genes with CNV, and analyze their RT and gene expression, all within the same cells. This will allow us to draw more detailed correlations between the three parameters and discover novel regulatory interactions between them.

We observed that the pseudo bulk RT profile derived from the mid-S phase sc-RT profiles had a high correlation to the HepG2 bulk RT profile. This allows us to drastically cut down on cell numbers compared with the bulk protocol. Here, we correlated RT with gene expression at the pseudo bulk level and the individual cell level. We were able to observe cell-specific RT-RNA correlations. These nuances could not be observed in the bulk plots and underscores the ability of the sc-multiomics approach to capture cell-specific differences.

While the protocol is compatible with cells or nuclei and can be used to process very small cell numbers, it is currently not high throughput. A future focus would be to customize the steps of the protocol to incorporate multiplexing and molecular barcoding to enhance high throughput. Another drawback is that we only gain insight into the mature RNA but not RNA without the poly(A) tail, such as ribosomal RNA, microRNA, transfer RNA, and long noncoding RNA. gDNA and RNA separation techniques, such as those used in scONE-Seq (35), can be performed upstream of the in-house gDNA and mRNA amplification, to ensure that the whole transcriptome is captured.

The HepG2 multiomics dataset generated in this study can be used to generate gene regulatory networks to dissect molecular interactions at the single-cell level. For example, we observed CNV in tumor suppressor genes (*TP53*), cell cycle regulator genes (*CDKN2A*), proto-oncogenes (*MYC*), genes involved in major pathways such as Wnt signaling (*CTNNB1*), PI3K/AKT pathway (*PTEN*), and others, all of which have been found to be mutated in HepG2 cancer cells as well as patient samples of hepatocellular carcinoma. This multiomics dataset can now be used to study CNVs of specific genes and observe their gene expression and downstream targets, all within the same cell. This dataset can also be combined with single-cell ATAC-Seq and Hi-C datasets to gain insight into higher level chromosomal organization and relate it to RT and gene expression. In this study, all HepG2 nuclei were processed by hand using multichannel pipettes. However, the protocol can also be automated and performed using liquid handling robots, such as EpMotion 5075, which will reduce manual processing time. An outline of the steps of the protocol is shown in Fig. S3, which can be used as a reference for automation of the robot. More detailed steps are described in the Experimental procedures section.

## Experimental procedures

### Sorting of HepG2 nuclei based on cell cycle phase

We isolated and stained HepG2 nuclei with a DNA-specific dye based on a previously optimized protocol (21). A total of 48 HepG2 nuclei were processed in this study from two different sorted plates (24 wells from each plate). The number of cells from each cell cycle phase gate is as follows—13 G1 phase cells, 6 S1 phase cells, 6 S2 phase cells, 5 S3 phase cells, 6 S4 phase cells, 6 S5 phase cells, and 6 G2 phase cells.

### Single-cell multiomics protocol for isolation and processing of gDNA and mRNA

The gDNA and cDNA were processed using the in-house multiomics protocol. Step-by-step description of the protocol is available at the protocol.io link in the *Data availability* section. We used the Nextera XT DNA Library Prep Kit for tagmentation-based library preparation of amplified gDNA and cDNA. To barcode each sample, we used unique oligos from the Nextera 96-index kit. All the gDNA and cDNA samples were then pooled on the same chip for sequencing. We used the NextSeq 2000 platform for sequencing—NextSeq P1 300 cycles, 150 PE. ∼two million reads for gDNA, and one million reads for cDNA were sufficient for deducing sc-RT and single-cell gene expression profiles.

### Sc-RT analysis from HepG2 single-cell gDNA

We used the Kronos scRT pipeline for generating the sc-RT binarized heatmaps. G1-sorted HepG2 cells were used as controls for normalizing gDNA reads from the S-phase HepG2 cells. We used bin sizes of 200 kb for generating the scRT profiles using the Kronos pipeline (8). We generated the scRT bed graphs showing nonbinarized individual cell trends as well as the pseudo bulk plots using the sc-Repli-Seq pipeline (23). For the scRT bed graphs, we counted reads in 200 kb sliding windows at 40 kb intervals and then normalized reads against the HepG2 G1 control cells.

For the pseudo bulk plot, we considered mid S phase sc-RT profiles of HepG2 nuclei (17 nuclei) from the S2, S3, and S4 sorting gates. The reads from S2 to S4 cells were combined and processed as a single entry using the sc-Repli-Seq pipeline (23). Reference bulk HepG2 RT data were obtained from Gene Expression Omnibus (GEO) dataset—GSM923446. CNV was calculated as part of the Kronos scRT pipeline by loading the function “CNV,” which considers chromosomes of interest, chromosome size, and genome wide bins. The CNV was adjusted per bin. Genes present in the bins that required adjustment are listed in the Supplemental file 1.

### Analysis of HepG2 gene expression and analysis of G1, S, and G2 markers

We used the Seurat v4 pipeline for the single-cell RNA analysis and for generating the heatmaps and clusterProfiler for the GO analysis. A Seurat object was created with the count data of the scRNA datasets. Cells with low genes/unique molecular identifiers were filtered out. Each cell was assigned to their specific cell cycle phase. For comparing phase-specific markers across G1, S, and G2 cell populations, S, G1, and G2 phase markers were loaded, and the scaled transcripts per million (TPM) normalized read counts (*Y*-axis) of these markers were plotted against the respective cell cycle phase (*X*-axis). Scaled TPM normalized counts were calculated as follows: scaled value = (x−μ)/σ; where x = TPM expression value, μ = mean expression across cells, and σ = standard deviation. Statistical significance between cell populations was calculated using the pairwise *t* test between phase groups. The stat_compare_means() was used for the statistical analysis, and default *p* values were used with a *p* ≤ 0.05 cutoff. For the heatmaps and GO analysis, DEGs between groups of G1 *versus* S and S *versus* G2 were identified using Seurat’s FindMarkers() function. The DESeq2 test method was used for differential expression analysis, which is specifically designed for RNA-Seq count data. Genes were filtered only to account for genes that were statistically different between groups (*p* < 0.05). From the list of DEGs, those that had scaled data in the Seurat object were then selected. The scaled top 100 DEGs between groups were plotted. GO term enrichment analysis was performed using the enrichGO() function, and the enriched terms were plotted in the barplots. Correlation between scRNA-Seq data generated in this study was performed with the reference scRNA dataset GEO—GSE90322. Genes with Ensembl ID were compared between the reference dataset, and the single cells were separated by cell cycle phases (G1, S, and G2). A list of the top 100 DEGs between cell cycle stages used in Figure 4 is included in Supplemental File 1.

### Correlation between RT and gene expression

For the pseudo bulk correlation between RT and gene expression, the pseudo bulk RT plot of S phase single cells was loaded and annotated by gene ID. The RT vector was joined with the corresponding RNA vector by gene ID. Genes with log_2_ Fragments Per Kilobase of transcript per Million mapped reads (FPKM) >1 in at least one cell were selected. Genes were then arranged by RT and subsequently binned together in groups of 50 genes. Within each bin, the expression level of genes was binarized (1 = expressed if log_2_FPKM >1, 0 = not expressed if log_2_FPKM ≤1), and subsequently, the probability of gene expression/bin of 50 genes was calculated and plotted against RT. Accordingly, the correlation line was computed, and the slope value was saved. A similar approach was used for deducing the bulk RT-RNA correlation plot. HepG2 bulk RT-RNA correlation was plotted using the reference bulk RT (GEO-GSM923446) and bulk RNA-Seq (GEO-GSM923446) datasets. Sc-RT plots were used for RT-RNA correlation. The probability of sc-gene expression (for genes with log_2_FPKM >1 and for bins of 50 genes) was plotted against sc-RT. The RT-RNA correlation slopes and Rep scores for individual cells are listed in Supplemental file 1.

## Data availability

• All raw and processed sequencing data generated in this study have been submitted to the National Center for Biotechnology Information GEO (https://www.ncbi.nlm.nih.gov/geo/) under accession number GSE283896.
• Code used for the analysis has been uploaded at the GitHub link—https://github.com/AnalaShetty1/HepG2-scripts.

• The detailed steps for the single-cell multiomics protocol are at protocols.io link—DOI: dx.doi.org/10.17504/protocols.io.36wgqdnwyvk5/v1.

## Supporting information

This article contains supporting information.

## Conflict of interest

The authors declare that they have no conflicts of interest with the contents of this article.

## Figures and Tables

**Supplemental Figure 3:**